# miR-210-3p suppresses osteogenic differentiation of MC3T3-E1 by targeting brain derived neurotrophic factor (BDNF)

**DOI:** 10.1186/s13018-022-03315-x

**Published:** 2022-09-14

**Authors:** Li Deng, Shuang Lai, Liyuan Fan, Xinlun Li, Hao Huang, Yandong Mu

**Affiliations:** 1 Stomatology Department, Sichuan Provincial People’s Hospital, School of Medicine, University of Electronic Science and Technology of China, Chengdu, 611731 China; 2 Institute of Tissue Engineering and Stem Cells, Nanchong Central Hospital, The Second Clinical College of North Sichuan Medical College, Nanchong, 637000 Sichuan China; 3 Stomatology Department, Sichuan Provincial People’s Hospital, Chengdu, 610072 Sichuan China; 4 Key Laboratory of Advanced Technologies of Materials (Ministry of Education), School of Materials Science and Engineering, Southwest Jiaotong University, Chengdu, 610031 Sichuan China

**Keywords:** miR-210-3p, Osteogenic differentiation, Cell migration, BDNF

## Abstract

**Background and objective:**

As an important mediator of intercellular interaction and formation of extracellular bone matrix, porous scaffolds are widely used for bone regeneration. Accumulating evidences demonstrate that microRNA are involved in the regulation of scaffolds-induced bone regeneration. Recently, we revealed that miR-210-3p was highly expressed during osteogenesis induced by HAG. In present study, we further explored the molecular mechanism underlying the effect of miR-210-3p on osteogenic differentiation.

**Materials and methods:**

In this study, miR-210-3p mimics and inhibitors were synthesized and transfected into MC3T3-E1 cells to explore their effects on osteogenic differentiation. The expression of osteogenic marker (Alp and Runx2) were detected by real-time quantitative PCR (qRT-PCR) and western blotting. After osteogenesis induction for 7 days, Alp staining were used to detected osteoblast differentiation of MC3T3-E1 cells. CCK8 and Transwell assays were performed to detected cell proliferation and migration. Then, top ranking list of target genes of miR-210-3p obtained from TargetScan and the expression of BDNF were detected by qRT-PCR and ELISA. The relationship between miR-210-3p and BDNF was verified by luciferase report assay. Furthermore,

the effect of BDNF on osteoblast differentiation was verified by transfecting siRNA or adding BDNF to the culture medium.

**Results:**

MiR-210-3p mimics markedly suppress osteogenic differentiation, cell migration and cell proliferation of MC3T3-E; nevertheless, silencing of miR-210-3p dramatically enhanced MC3T3-E1 osteogenesis, cell migration and proliferation. Furthermore, luciferase reporter assay verified that brain derived neurotrophic factor (BDNF) is a directly target of miR-210-3p. Moreover, BDNF siRNA significantly decreased the expression levels of ALP and cell migration. The addition of BDNF partially rescued the inhibition of osteogenesis by miR-210-3p.

**Conclusion:**

miR-210-3p inhibited the osteogenic differentiation via targeting BDNF. Our Results provide a promising target for regulating osteogenic differentiation.

## Introduction

Bone defects caused by different causes lower people's quality of life and increase economic burden. Despite the regenerative potential of bone, the repair of large bone defects is still a major clinical problem. Autografts, as the gold standard for bone defect repair, has disadvantages such as limited bone graft volume, potential donor site lesions and wound infection [1–3]. Therefore, bone tissue engineering is widely used in bone regeneration as the most promising method. Scaffold is an important part of bone tissue engineering. In previous study, we developed a grooved porous hydroxyapatite scaffold (HAG) with good osteogenic effect in vitro and in vivo [4].

As a mineralized mesenchymal tissue, bone regeneration is a complex process involving the balance of various cell differentiation [5]. This process is extensively regulated. MicroRNAs (miRNAs), a group of non-coding RNA consisting of 20–24 nucleotides are evolutionarily conserved, interaction of target mRNA sequences, negative regulation of their expression and participate in biological processes including cell differentiation and proliferation [6–8]. At present, they have been confirmed to be tightly involved in the regulation of bone formation by targeting different target genes and negatively regulating their expression [9]. MiR-23a, miR-30c, miR-34c and miR-133a et al. have been confirmed that significantly inhibit osteoblast differentiation by targeting RUNX2 (RUNX family transcription factor 2, an essential regulatory factor for skeletal morphogenesis) [10]. MiR-21 promotes BM-MSCs osteogenesis by targeting an TGF signal antagonist SMAD7 [11]. Upon the development of bone tissue engineering, the important role of miRNA in scaffold osteogenesis is increasingly being revealed [12–14]. Maryam Izadpanahi et al. investigated the nanorographic niche interactions with of non-coding RNA in stem cell fate to reveal deeper mechanisms for enhancing osteogenic differentiation. They found that the activity of miRNA in hMSCs was closely related to the nanotopographical cues and that the nanotopographical cues regulated the osteogenic differentiation of hMSCs via modulating lncRNAs [15]. Recently, we found that miRNAs were differentially expressed during osteogenesis induced by HAG scaffolds, and miR-210-3p was one of the significantly up-regulated miRNAs [16]. However, its role of osteogenic differentiation remains unclear.

In the present study, the role and mechanism of miR-210-3p in the osteogenic differentiation were investigated. Our findings could provide new insights and target for the diagnosis and treatment of bone regeneration.

## Materuials and methods

### Cell culture and transfection

MC3T3-E1 cell line were obtained from the Cell Bank of Typical Culture Preservation Committee of the Chinese Academy of Science, Shanghai, China. Cells were cultured in complete medium (MEM (Hyclone) with 10% fetal bovine serum (FBS, Avantor®) and 1% penicillin streptomycin (HyClone)).

MiR-210-3p mimic, inhibitor and BDNF siRNA (synthesized by GenePharma, 100 pM) were transfected when the cell density approached 80% using the Lipofectamine™ 2000 (Thermo Fisher Scientific) transfection reagent following the manufacturer’s protocol. After 24 h, the medium was replaced with osteogenic induction medium. 20 mM β-glycerophosphate and 50 g/mL vitamin C were added to complete medium for osteogenic induction. Change the medium every three days. After induction for 7 days, Alkaline phosphatase (ALP) expression was detected according to the manufacturer’s instructions (TRAP/ALP Stain Kit, Wako).

### Quantitative real-time PCR

TRIzol™ reagent (Invitrogen) was used for total RNA extraction. M-MLV (Thermo Fisher Scientific) was used for reverse transcription of RNA. Realtime PCR were performed by ABI 7500 (with 2X ChamQ Universal SYBR Master Mix (Vazyme, China)). The primer sequences used are as follows:

**Table:** 

Gene	Forward (5′-3′)	Reverse (5′-3′)
miR-210-3p	ACTGTGCGTGTGACAGC	GAGAGGAGAGGAAGAGGGAA
Alp	GCAGTATGAATTGAATCGGAACAAC	ATGGCCTGGTCCATCTCCAC
Gapdh	AGGTCGGTGTGAACGGATTTG	TGTAGACCATGTAGTTGAGGTCA

### Protein extraction and western blot analysis

Total protein was extracted using RIPA lysis buffer (Beyotime), and the proteins were quantitatively determined by BCA kit (Biosharp). 10% SDS-PAGE gel electrophoresis was used to separate protein samples for Western blot analysis. The primary antibodies used were anti-ALP (HuaBio, 1:2000), Runt-related transcription factor 2 (RUNX2) (HuaBio, 1:2000), GAPDH (HuaBio, 1:5000). The HRP-conjugated secondary antibody was used to enable detection. The super-ECL detection system (Biosharp) was used in combination with primary antibody.

Enzyme-Linked Immunosorbent Assay (ELISA).

The Mouse BDNF (Brain Derived Neurotrophic Factor) ELISA Kit (Elabscience) was used to measure the protein level of BDNF in the medium. The culture supernatants were collected and centrifuged for 20 min at 1000X g. Then processed according to the manufacturer’s instructions and measured the absorbance at 450 nm.

### Cell migration and proliferation

Transwell chambers (8 μm pores, Corning) were used to detect cell migration. In details, the MEM cell suspension (5 × 10^4^ cells) was seeded in the chamber and the lower filled with complete medium. After 24 h and 48 h of culture, the MEM in the chamber was discarded, and cotton wiped cells from the upper chamber. The bottom cells were stained with crystal violet after fixation with 4% PFA for 15 min. Finally, the cells were photographed and analyzed by image J. For proliferation detected, cells were seeded in 96-well plate after transfection. According to manufacturer’s protocol, a CCK8 assay (Solarbio) was performed on day 3 and 5. An absorbance of 450 nm represents the cell proliferation ability. The experiments were repeated three times.

### Dual luciferase reporter assay

Target scan predicted the 3' untranslated region (UTR) binding site of BDNF to miR-210-3p. Luciferase assays were performed to verify the interaction between miR-210-3p and BDNF. Briefly, BDNF luciferase reporter plasmid (constructed by GenePharma) was transfected into MC3T3-E1 cells with miR-NC or miR-210-3p mimic. The medium was replaced after 4 h. After incubated for 24 h, a Dual-Luciferase Reporter Test Kit (Vazyme) was used to measure the Firefly and Renilla luciferase activities. The ratio of firefly to Renilla luciferase activity is the relative luciferase activity of each sample. The experiments were repeated three times.

### Statistical analysis

GraphPad Prism 8 was used for statistical analyses. Student’s t test was conducted to compare means between two groups. All data are expressed as the mean ± standard deviation. *P* values < 0.05 were considered statistically significant.

## Results

### miR-210-3p inhibited the osteogenic differentiation of MC3T3-E1 cells

**Fig. 1:**
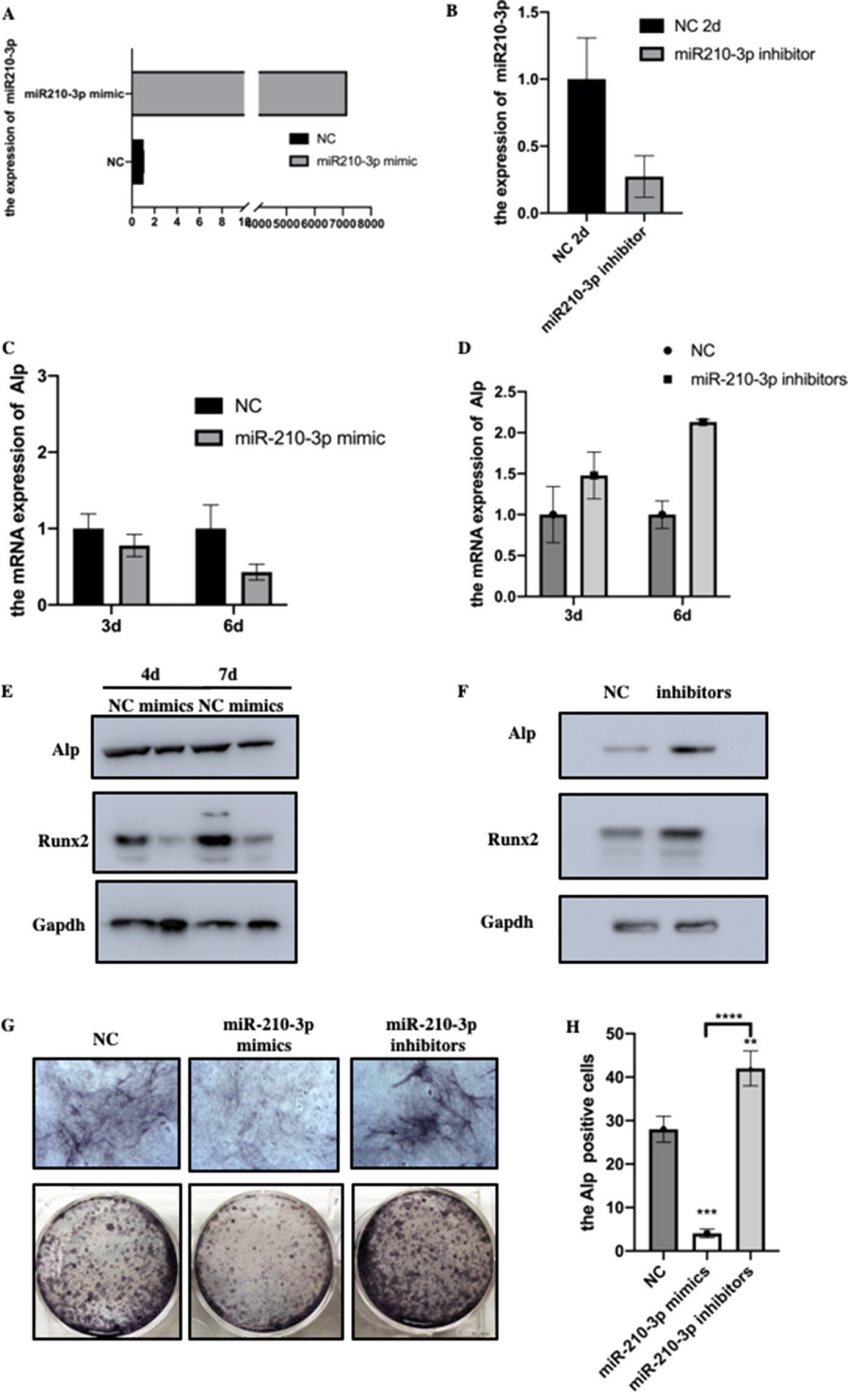
miR-210-3p inhibited the osteogenic differentiation of MC3T3-E1 cells. **A** and **B** Expression of miR-210-3p after transfection with mimics or inhibitor were examined by qRCR; **C** and **D** The mRNA expression of Alp was detected after transfection with mimics or inhibitor and osteogenesis induction for 3 days and 6 days; **E** and **F** The protein expression of Alp and Runx2 were detected after transfection with mimics or inhibitor; **G** Representative images of Alp staining after transfection and Columnar analysis diagram (H)

Our previous study revealed that miR-210-3p was upregulated in HAG scaffold induced osteogenic differentiation [16]. miR-210-3p is a highly conserved miRNA from mouse to humans (sequence: CUGUGCGUGUGACAGCGGCUGA). For uncovering the role of miR-210-3p in osteogenic differentiation, we first achieved efficient overexpression or silencing of miR-210-3p in cells by transfection, as shown in Fig. 1A and B. Then, we further detected osteogenic markers expression. Surprisingly, the results showed that miR-210-3p overexpression decreased Alp mRNA expression, while miR-210-3p inhibitors increased it (Fig. 1C and D). Consistently, the protein expression of Alp and Runx2 were decreased or increased with miR-210-3p mimic or inhibitor transfection (Fig. 1E and F). Moreover, ALP staining also showed that miR-210-3p mimic significantly decreased ALP expression while inhibitor promoted (Fig. 1G and H). These results suggested that miR-210-3p inhibited the osteogenic differentiation of MC3T3-E1 cells.

### miR-210-3p inhibited the proliferation and migration of MC3T3-E1 cells

**Fig. 2:**
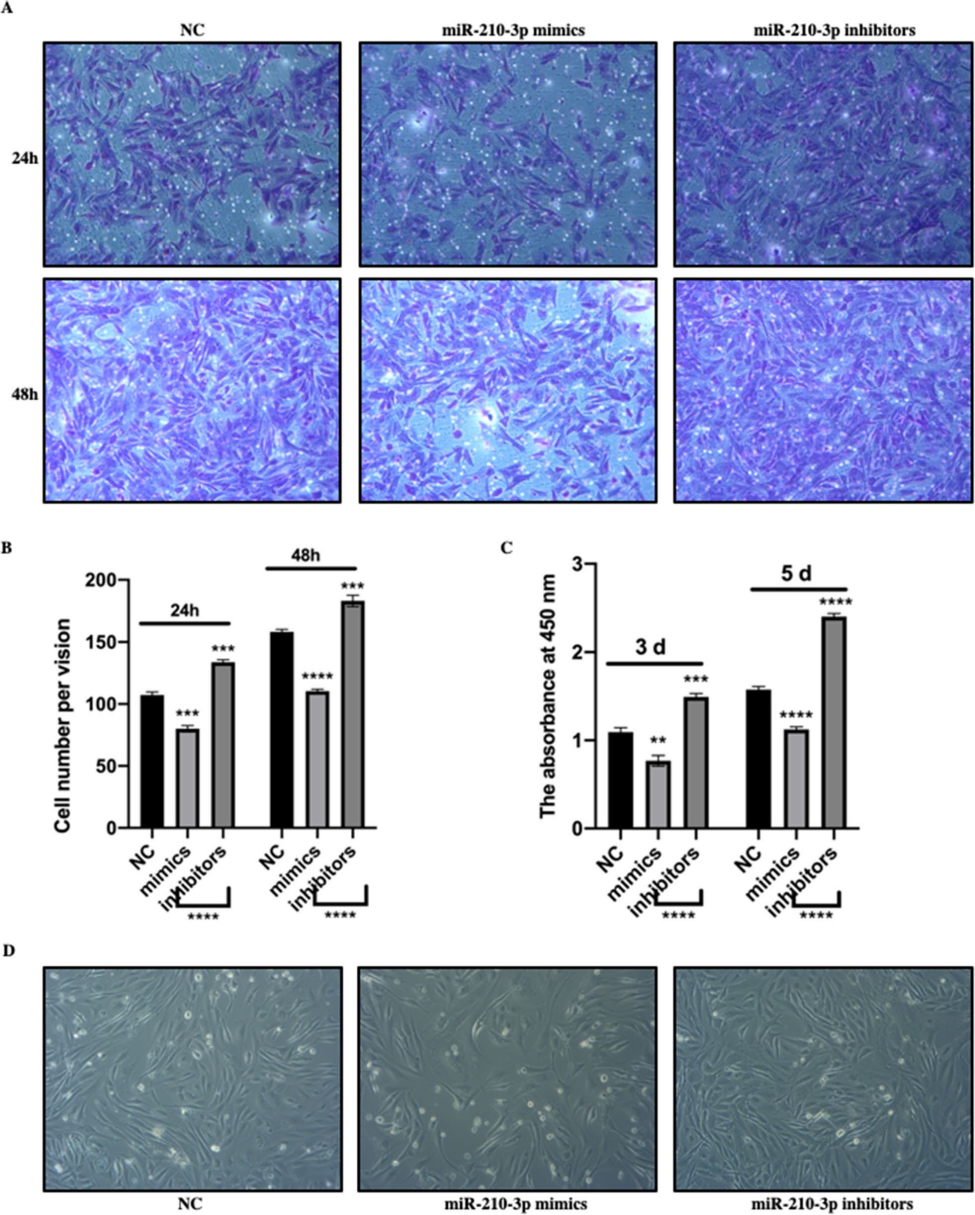
miR-210-3p inhibited the proliferation and migration of MC3T3-E1 cells. **A** The migration of MC3T3-E1 cells transfected with miR-210-3p mimic or inhibitor was evaluated, assessed by staining, photographed and Columnar analysis diagram (**B**). **C**. The proliferation of MC3T3-E1 cells transfected with miR-210-3p mimic or inhibitor was detected by CCK-8 assay and cell images at day 5 (**D**)

Cell proliferation and migration have been proved to be closely related to their osteogenic differentiation ability. Here, cell migration of MC3T3-E1 was detected after transfection with miR-210-3p mimic or inhibitor. As shown in Fig. 2A and B, crystal violet staining showed that miR-210-3p overexpression inhibited the migration of MC3T3-E1 cells cultured 24 h or 48 h in Transwell chambers, while inhibitors promoted. Similarily, the results of CCK8 assay showed that highly expression of miR-210-3p reduced cell proliferation of MC3T3-E1 cells (Fig. 2C). Cell images were photoed on the fifth day after culture and showed that miR-210-3p significantly negatively regulated cell proliferation (Fig. 2D).

### BDNF is a directly target of miR-210-3p

**Fig. 3:**
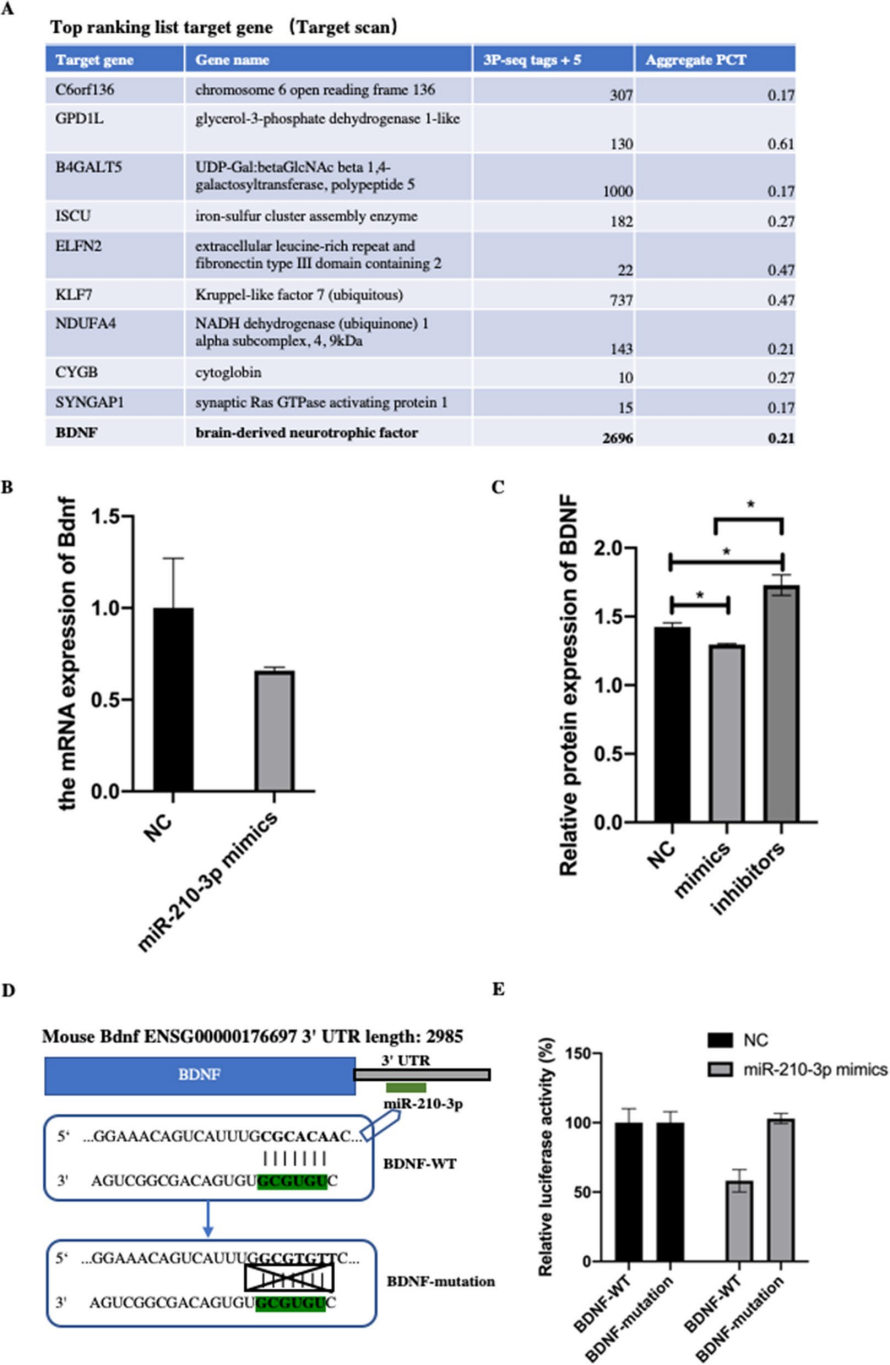
BDNF is a directly target of miR-210-3p. **A** Top ranking list of miR-210-3p target genes obtained from TargetScan software. **B** The mRNA expression of Bdnf after transfection with miR-210-3p mimics were examined by qRCR. **C** Relative protein expression of Bdnf were examined after transfection with miR-210-3p mimics or inhibitor. **D** Binding sites of miR-210-3p and the BDNF 3′UTR, as detected by luciferase reporter assays. BDNF mutation: luciferase reporter plasmid containing mutant BDNF 3′UTR. BDNF -WT: luciferase reporter plasmid containing wild-type BDNF 3′UTR. E Relative luciferase activities of luciferase reporters containing WT or MUT BDNF 3′-UTR in MC3T3-E1 cells transfected with miR-210-3p mimic

To uncover the downstream molecular mechanism contributing to miR-210-3p mediated osteogenesis, TargetScan software was used to identify potential targets of miR-210-3p. Notably, we found that BDNF as an important target of miR-210-3p (Fig. 3A). Furthermore, we detected the mRNA and protein expression of Bdnf after miR-210-3p transfection to validate the prediction. Indeed, as shown in Fig. 3B and C, Bdnf decreased after miR-210-3p overexpression, while increased after silencing. Moreover, we constructed the BDNF luciferase reporter plasmid, which conained either wild type fragments (BDNF-wt) or mutant binding site fragments (BDMF-mut) downstream of the firefly sequence (Fig. 3D). The miR-210-3p mimic was co-transfected into MC3T3-E1 cells with the constructed reporter plasmids. The results showed that compared to BDNF-mut, co-transfection of BDNF-wt with miR-210-3p mimic decreased luciferase activity (Fig. 3E). These data imply that BDNF is a directly target of miR-210-3p.

### BDNF promoted osteoblast differentiation, cell migration and partially rescued the miR-210-3p mediated inhibition of osteogenesis

**Fig. 4:**
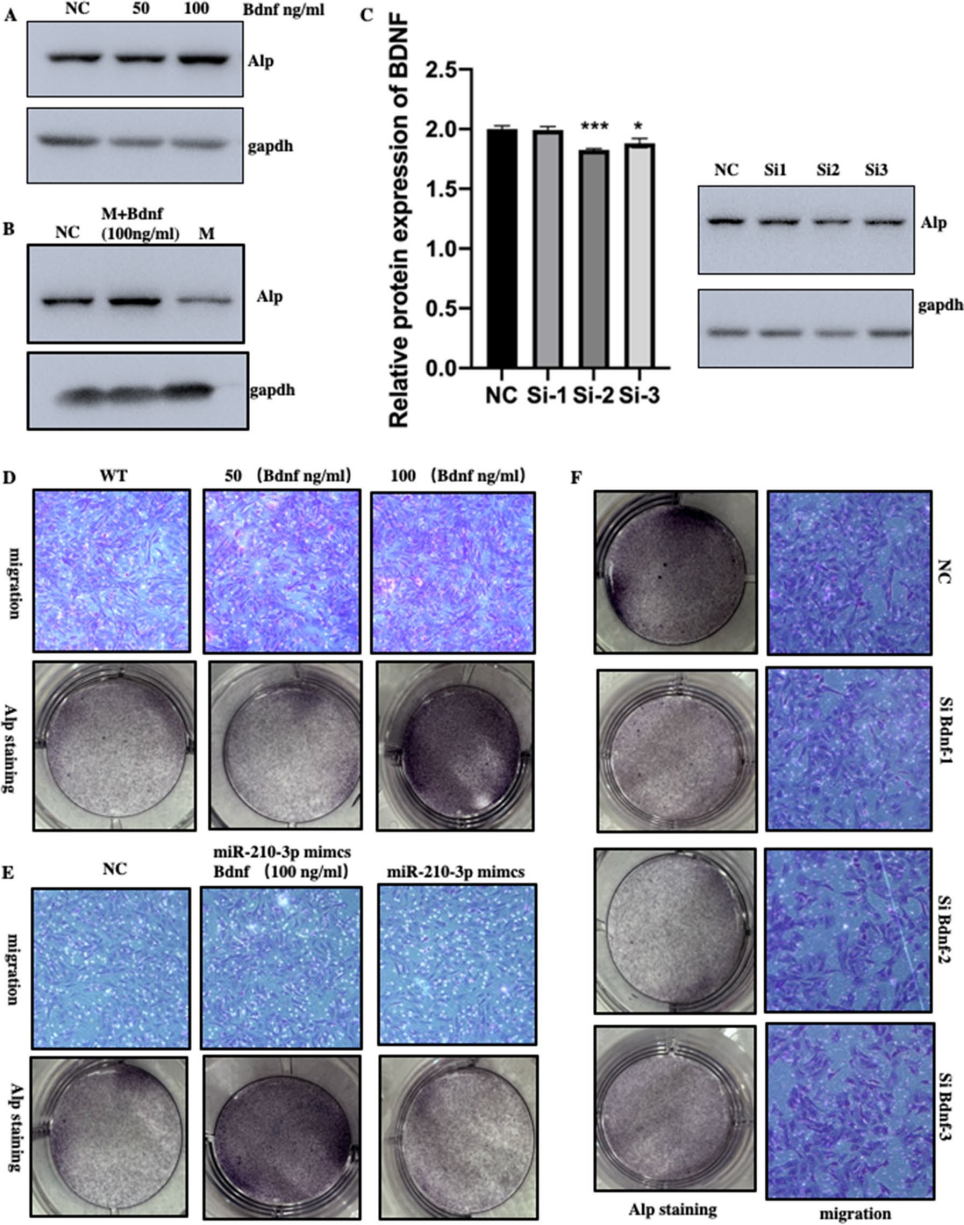
BDNF promoted osteoblast differentiation, cell migration and partially rescued the miR-210-3p mediated inhibition of osteogenesis. **A** and **B** Western blot analysis of protein expression of Alp (A. BDNF added; B. BDNF rescue). **C** Relative protein expression of BDNF and Alp were examined after transfection with BDNF siRNA. **D** and **F**. The images of cell migration and Alp staining (D. BDNF added; E. BDNF rescue; F. BDNF siRNA transfection)

In order to further reveal the role of miR-210-3p and BDNF in osteogenic differentiation of MC3T3-E1 cells, we first added different concentrations of BDNF in the medium to induce osteogenic differentiation. Western blotting showed that BDNF-added increased the expression of ALP (Fig. 4A), and the BDNF-added could partially rescue inhibitory effect of miR-210-3p on ALP expression (Fig. 4B). Furthermore, transfection of BDNF siRNA silenced the expression of BDNF and decreased the expression of Alp (Fig. 4C). The promoting effect of BDNF on MC3T3-E1 cell proliferation and ALP expression was also confirmed by ALP staining and cell migration assay ALP staining and transwell assay also showed that BDNF could promote the osteogenic differentiation and cell migration of MC3T3-E1 cells (Fig. 4D). Meanwhile, the BDNF-added also could partially rescue the inhibitory effect of miR-210-3p on ALP expression and cell migration in MC3T3-E1 (Fig. 4E). Consistently, the silencing of BDNF inhibits ALP expression and cell migration of MC3T3-E1 cells (Fig. 4F). These data confirmed that miR-210-3p inhibited the osteogenic differentiation of MC3T3-E1 cells by targeting BDNF.

## Discussion

The osteogenic differentiation ability of cells is essential for bone regeneration. Increasing evidences suggest that miRNAs are important and functional in regulating osteogenesis [17, 18]. Hassan et al. and Zhang et al. found that miR-218 has positively effects on the Wnt signaling pathway, through targeted the signaling different inhibitors, including DKK2, SFRP2 and SOST [19, 20]. Meanwhile, SOST also was targeted and inhibited by miR-96 [21]. MiR-146a was found to negatively regulate osteogenesis and bone regeneration both in vitro and in vivo through interaction with SMAD4 [22]. However, the role of miR-210-3p in osteogenic differentiation remains unclear. In current study, our data exhibit that miR-210-3p obviously inhibited the osteogenic differentiation and cell migration of MC3T3-E1 cells by targeting BDNF.

After bone graft, the loss of neuralized vascular network in the implanting area would affect subsequent bone regeneration. Brain-derived neurotrophic factor (BDNF) is a widely recognized growth factor involved in the regulation of neuronal growth, survival and angiogenesis [23]. Moreover, BDNF seems to be considered a growth factor suitable for bone material implantation. Yamashiro et al. and Kilian et al. showed that the mRNA and protein of BDNF existed in osteoblasts and were increased during human fracture healing [24]. In addition, the role of BDNF in promoting osteogenic differentiation has been confirmed both in vitro and in vivo [25, 26]. Consistently, in our study, we confirmed that BDNF promoted the osteogenic differentiation and cell migration of MC3T3-E1. Furthermore, the addition of BDNF can partially rescue the inhibition caused by miR-210-3p.

Recently, miRNA has also been shown to play a vital regulatory role in tendon healing and osteoarthritis (OA), involving apoptosis, senescence of tendon stem/progenitor cell, and maintenance of chondrocyte homeostasis [27, 28]. Furthermore, based on the natural processes of regulation of eukaryotic genes. Small interfering RNAs (siRNAs) mediated gene silencing has been used to regulate tendon homeostasis and human rheumatoid arthritis [29, 30]. Consistently, in present study, the functional role of miR-210-3p may introduce the idea of using miR-210-3p targeted silencing-modified cell or scaffolds for bone repair. Meanwhile, the expression of miR-210-3p may be a prognostic indicator of bone regeneration. Moreover, BDNF transfer may be an efficient way to bone regeneration through regulate bone metabolism and promote osteogenesis.

In conclusion, our study has shown a clear link between miR-210-3p and BDNF in MC3T3-E1 cells and demonstrated that miR-210-3p inhibited osteogenic differentiation by targeting BDNF. This suggests that silencing miR-210-3p or overexpressing BDNF may have a future role in improving bone tissue engineering.

## Data Availability

The data that support the findings of this study are available on request from the corresponding author.
